# Unusual Cytology Features of A *DICER1* ‐Mutated Thyroid Nodule: Raising Awareness of A Potential Diagnostic Pitfall During Fine Needle Aspiration

**DOI:** 10.1111/cyt.70053

**Published:** 2026-01-20

**Authors:** Wenli Dai, Eduardo V. Zambrano, Sabri Yilmaz, Qian Wang

**Affiliations:** ^1^ Department of Pathology University of Pittsburgh Medical Center Pittsburgh Pennsylvania USA; ^2^ School of Medicine University of Pittsburgh Pittsburgh Pennsylvania USA; ^3^ Department of Pathology and Laboratory Medicine Phoenix Children's Hospital Phoenix Arizona USA; ^4^ Department of Child Health University of Arizona College of Medicine Phoenix Arizona USA; ^5^ Department of Pathology Creighton University School of Medicine Phoenix Arizona USA; ^6^ Department of Radiology UPMC Children's Hospital of Pittsburgh Pittsburgh Pennsylvania USA; ^7^ Department of Pathology UPMC Children's Hospital of Pittsburgh Pittsburgh Pennsylvania USA

**Keywords:** *DICER1*, FNA, infarction, thyroid

## Abstract

*DICER1*‐mutated thyroid lesions can range from indolent to aggressive neoplasms. Infarction has been recently described as a histologic feature of *DICER1*‐mutated thyroid nodules. In this case report, we presented a *DICER1*‐mutated nodule which presented predominantly with debris in the fine needle aspiration (FNA) specimen and was confirmed on surgical resection to be a near total infarcted nodule. To the best of our knowledge, this is the first cytopathology report of a *DICER1*‐mutated thyroid lesion presenting as an infarcted nodule. Awareness of the features described herein may help prevent both overdiagnosis and underdiagnosis in the evaluation of thyroid nodules via FNA.

## Graphical abstract

This article describes the unusual cytology features of a *DICER1*‐mutated thyroid nodule. Fine needle aspiration revealed predominantly cell debris/degenerated cells and rare viable follicular cells with mild cytologic atypia. Surgical resection revealed a nodule with nearly complete infarction, which is one specific feature of *DICER1*‐mutated thyroid nodules.*DICER1*‐ mutated thyroid nodule may present with an infarct nodule, which is a potential diagnostic pitfall for fine needle aspiration (FNA).

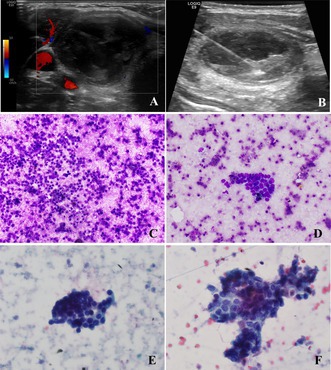

## 1 Introduction

*DICER1*‐gene encodes an RNase III endoribonuclease essential for the cleavage of pre‐microRNA to mature microRNA, and *DICER1* disruption/mutation results in alteration of miRNA transcripts in cancers [1]. Somatic and germline *DICER1* mutations are reported in subsets of thyroid tumours, supporting the role of this gene in thyroid tumour development. There is a broad spectrum of associated thyroid lesions, including follicular adenomas, follicular carcinomas, papillary thyroid carcinomas, and more rarely paediatric poorly differentiated thyroid carcinoma and thyroblastoma, demonstrating the range of indolent to aggressive neoplasms [2]. Recently, specific histologic features have been described with *DICER1*‐mutated thyroid nodules, including macrofollicular structures, abortive areas or infarction, and focal papillary growth. Infarction is possibly a highly sensitive and specific feature associated with *DICER1*‐mutated thyroid nodules [3, 4, 5]. While previous studies have reported microfollicular or crowded component, and papillary excrescences in FNA samples [6, 7, 8], to the best of our knowledge infarction has never been reported in cytology specimens. Herein, we report a thyroid nodule which presented with near total infarction at initial FNA biopsy.

## 2 Case Report

**FIGURE 1:**
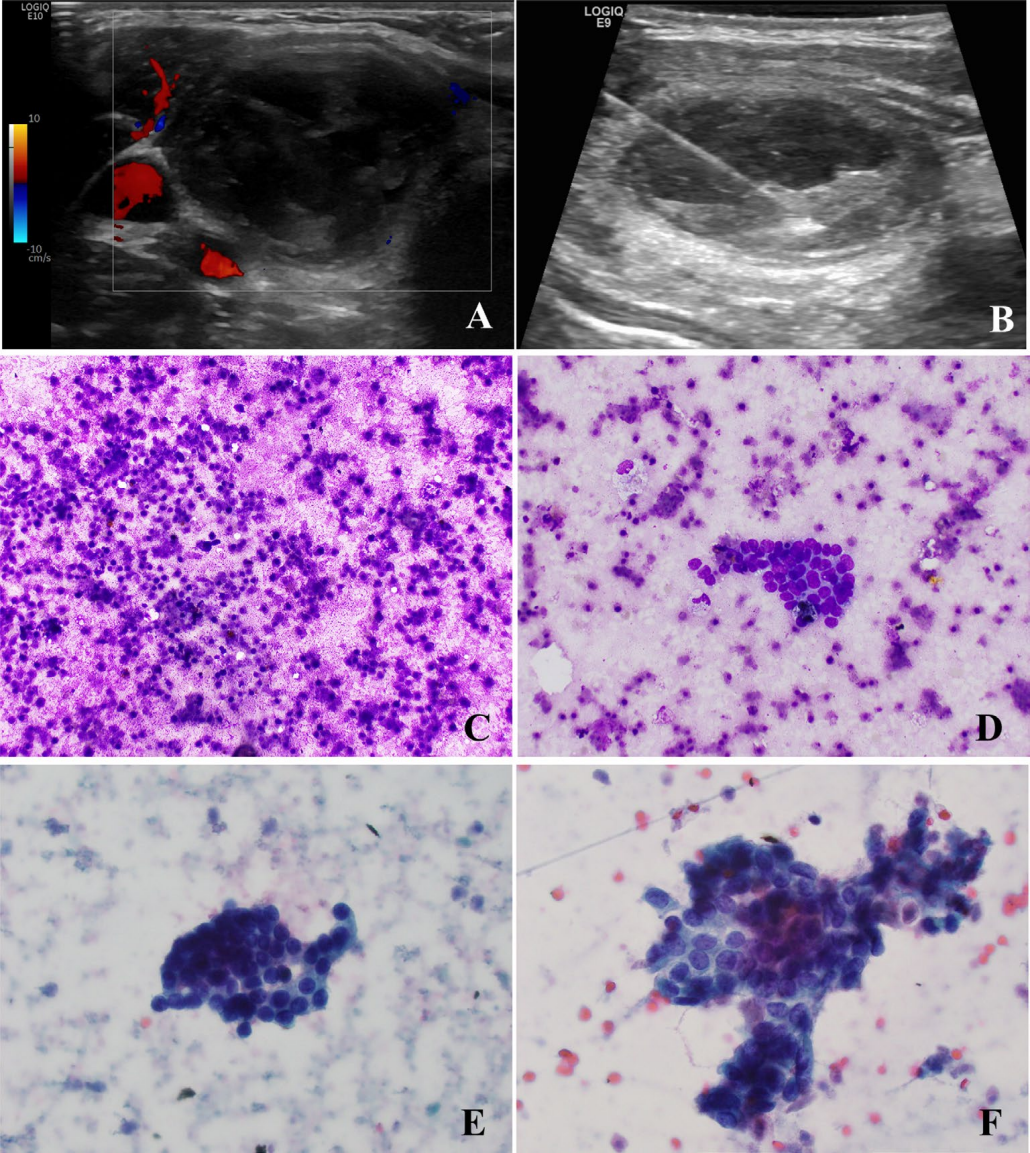
FNA of the thyroid nodule showed predominant cell debris with rare viable thyroid follicular cells. (A) Colour ultrasound evaluation showed no internal blood flow of a 3.6 cm × 2.8 cm × 2.2 cm ovoid, well‐defined, and heterogeneous but mostly hypoechoic lesion in the inferior portion of the right thyroid gland. This lesion had smooth contours and no calcifications within the lesion or its periphery. (B) Real time ultrasound image shows fine needle aspiration biopsy with a 25‐gauge needle. (C) Diff‐Quik stain showed abundant degenerated cells and debris (20×). (D) Diff‐Quik stain showed rare viable follicular cells (20×). (E and F) PAP stain showed rare viable follicular cells with mild cytologically atypia, including slightly enlarged and mildly pale nuclei with focally nuclear crowding (40×).

The patient was a 15‐year‐old female who presented to the emergency department with mildly painful right anterior neck swelling for 2 days with increasing size, mild difficulty swallowing and increased fatigue. Ultrasound demonstrated a 3.6 × 2.8 × 2.2 cm well‐defined, heterogeneous, avascular lesion, without calcification, in the right thyroid lobe (Figure 1A,B). TI‐RADS was not given. No suspicious lymph nodes were identified, and the remaining thyroid gland appeared normal. She had a family history of thyroid nodules in both maternal and paternal lines and numerous maternal family members with cancer histories; otherwise, she did not have a significant past medical history.

An ultrasound‐guided FNA was performed. Five total passes with 25 G needles were performed. Only the last pass was with positive aspiration for cell block preparation. The aspiration fluid was slightly cloudy. Five alcohol‐fixed and five air‐dried direct smears were prepared from a total of five passes. Immediate interpretation of an air‐dried, Diff‐Quik stained smear from each pass was performed to assess adequacy. The remaining alcohol‐fixed smears were Papanicolaou stained. The smears showed a specimen composed of rare clusters of viable follicular cells in a background of abundant degenerated/ghost cells (Figure 1C) with intermingled rare neutrophils and lymphocytes, foamy and hemosiderin‐laden macrophages, and a small amount of watery colloid. The viable follicular cells showed mild cytological atypia characterised by slightly enlarged and mildly pale, focally crowding nuclei (Figure 1D–F). The cell block showed predominantly degenerated cell debris with only a few macrophages (highlighted by CD163 stain) and lymphocytes (highlighted by CD45). Overall, the findings were classified as atypia of undetermined significance (AUS).

ThyroSeq analysis of the specimen showed a *DICER1* p.E1813K, c.5437G>A mutation with a variant allele frequency of 41%. No other mutations, fusions, or copy number alterations were identified. No germline test was performed on this patient.

**FIGURE 2:**
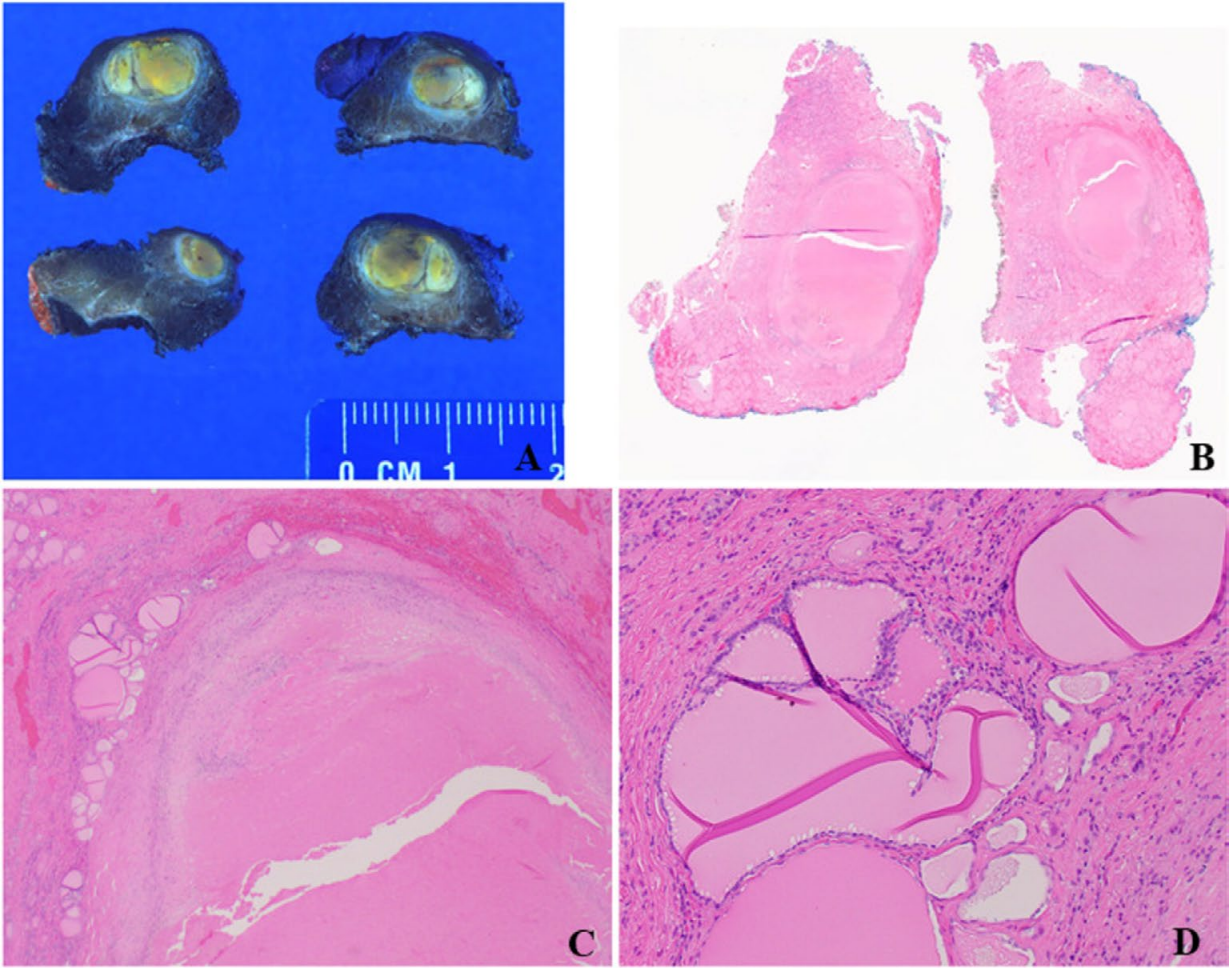
Right thyroid lobectomy specimen of the patient. (A) A gross examination showed a well‐circumscribed nodule with yellow and white coloration. (B) A scan magnification showed the nodule with near complete infarct. (C) A Low‐power magnification showed a nearly infarcted nodule, with residual viable cells present at the periphery. Histiocytes and scattered inflammatory cells were also observed at the edge of the nodule (2×). Histiocytes and scattered inflammatory cells were also present at the edge of the nodule (2×). (D) A higher‐power magnification showed the residual, bland looking thyroid follicles (10×).

A right thyroid lobectomy was performed, and a well circumscribed 1.2 cm nodule was found (Figure 2A). Extensive ischemic necrosis/infarction, with peripheral histiocytes/macrophages and only rare viable bland looking neoplastic follicles were present (Figure 2B,C). No evidence of malignancy was identified, including negative immunostains for HBME‐1 and Galectin‐3. Dystrophic microcalcifications without lamellations were present, mainly at the periphery of the nodule.

At most recent 1‐year follow‐up following surgery, T4, TSH, and thyroglobulin levels were within normal limits, and the patient was in good state of health.

## 3 Discussion

*DICER1* plays a central role in the biogenesis of microRNAs and is important for normal development. Altered microRNA expression and *DICER1* dysregulation have been described in several types of tumours. *DICER1* genetic alterations typically functions as either a tumour suppressor gene, resulting from loss‐of‐function mutations, or as an oncogene, stemming from gain‐of‐function mutations [1]. Common somatic mutation sites include hotspot amino acids 1705, 1709, 1809, 1810, and 1813, which are important for metal‐ion binding, a catalyst for the RNase activity [2].

Infarction has been described recently in *DICER1*‐mutated thyroid nodules, the mechanism of which is not disclosed. Trauma such as FNA may trigger infarction in thyroid nodules; however, our patient was not previously biopsied and the nodule infarction seemingly occurred prior to FNA, since FNA specimen demonstrated predominantly debris in all five passes. Therefore, it is likely that infarction of this *DICER1‐*mutated thyroid nodule was secondary to an intrinsic mechanism, which may also explain why *DICER1‐*mutated thyroid nodules have a higher frequency of infarction compared with *DICER1*‐wild‐type thyroid nodules [4].

Infarct in our FNA case presented with abundant cell debris with a few scattered lymphocytes and rare viable thyroid follicular cells, which can be a pitfall for tumour necrosis in high‐grade thyroid carcinomas. Our FNA biopsy showed a paucicellular specimen with rare viable follicular cells which had only mild cytological atypia. These features did not fit high‐grade thyroid carcinoma. Thyroiditis or intrathyroidal thyroglossal duct cyst were also considered in the differential diagnosis, as they may also show similar cytologic features such as abundant cell debris, inflammatory cells, and no/rare follicular cells.

*DICER1* mutations are rare in adult thyroid tumours, found in only 1.4% of fine‐needle aspiration cases [9]. However, these mutations are more common in paediatric thyroid tumours, with a frequency ranging from 20% to 50% [10]. Our case exemplifies a *DICER1*‐mutated thyroid nodule with unusual cytologic features on the initial FNA. Awareness of the features herein described can help to avoid over or under diagnosing these lesions, especially in paediatric patients, in which *DICER1* mutations are not uncommon. This case also highlights the crucial role that molecular genetic analysis, particularly next generation sequencing (NGS), plays in providing an accurate diagnosis of thyroid neoplasms.

## Author Contributions

**Wenli Dai:** collecting data and drafting the manuscript; **Eduardo V. Zambrano:** interpreting the data and editing the manuscript; **Sabri Yilmaz:** interpreting and preparing the radiological data; **Qian Wang:** designing and drafting the manuscript, interpreting the data, preparing the figures.

## Funding

The authors have nothing to report.

## Ethics Statement

The study was performed with Institutional Review Board approval.

## Consent

The authors have nothing to report.

## Conflicts of Interest

The authors declare no conflicts of interest.

## Data Availability

Data sharing not applicable to this article as no datasets were generated or analysed during the current study.
